# High Prevalence of aCL-IgA and aβ2GPI-IgA in Drug-Free Schizophrenia Patients: Evidence of a Potential Autoimmune Link

**DOI:** 10.3390/antib13040092

**Published:** 2024-11-15

**Authors:** Samar Samoud, Imen Zamali, Fatma Korbi, Ahlem Mtiraoui, Ahlem Ben Hmid, Neila Hannachi, Yousr Galai, Hechmi Louzir, Yousri El Kissi

**Affiliations:** 1 Department of Clinical Immunology, Institut Pasteur de Tunis, Tunis 1002, Tunisia; imen.zamali@fmt.utm.tn (I.Z.); ahlem.benhmid@pasteur.tn (A.B.H.); yousr.galai@pasteur.tn (Y.G.); 2 Faculty of Medicine of Sousse, University of Sousse, Sousse 4002, Tunisia; ahlamtiraoui@yahoo.fr (A.M.); naila.hannachi@rns.tn (N.H.); yousri.elkissi@yahoo.com (Y.E.K.); 3 Laboratory of Transmission, Control and Immunobiology of Infections (LR16IPT02), Institut Pasteur de Tunis, Tunis 1002, Tunisia; hechmi.louzir@pasteur.tn; 4 Faculty of Medicine of Tunis, University of Tunis El Manar, Tunis 1068, Tunisia; fatma.korbi@uneos.fr; 5 Laboratoire de Biologie Médicale, UNEOS, 57070 Metz, France; 6 Department of Psychiatry, Farhat Hached University Hospital, Ibn El Jazzar Street, Sousse 4000, Tunisia; 7 Research Laboratory LR12ES04, Faculty of Medicine of Sousse, University of Sousse, Sousse 4002, Tunisia; 8 Faculty of Pharmacy, University of Monastir, Monastir 5000, Tunisia

**Keywords:** schizophrenia, antiphospholipid antibodies, anticardiolipin, anti-beta-2 glycoprotein I, autoimmunity, IgA

## Abstract

Background/Objectives: Schizophrenia (SZ) is a complex psychiatric disorder with increasing evidence pointing to an autoimmune component, including the presence of antiphospholipid antibodies (aPLs). This study aims to assess the prevalence of anticardiolipin (aCL) and anti-beta-2 glycoprotein I (aβ2GPI) antibodies, particularly the IgG, IgA, and IgM isotypes, in drug-free SZ patients compared to healthy controls, and explore their possible involvement in the disease’s pathophysiology. Methods: Eighty SZ patients meeting DSM-IV criteria were recruited, along with 80 matched healthy controls. Serum samples were analyzed using enzyme-linked immunosorbent assays (ELISA) to quantify IgG, IgA, and IgM isotypes of aCL and aβ2GPI. Results: SZ patients exhibited significantly higher levels of aCL-IgM and aCL-IgA (*p* < 0.05), as well as elevated aβ2GPI-IgA (22.5%, *p* < 0.001), compared to controls. No significant differences were observed in the aCL-IgG isotype. Interestingly, 72% of aPL-positive SZ patients were positive for aβ2GPI-IgA, with some also co-expressing multiple isotypes, suggesting a potential link between SZ and antiphospholipid syndrome (APS). Conclusions: This study is the first to report a high prevalence of aCL-IgA and aβ2GPI-IgA in SZ patients, highlighting a possible autoimmune involvement in the disease. The presence of multiple aPL isotypes, particularly IgA, suggests a need for further investigation into their role in SZ pathogenesis and their potential association with APS.

## 1. Introduction

Schizophrenia is a major mental disorder with no clearly identified pathophysiology despite decades of research. Increasing evidence points to an immunological involvement in this disease, particularly through the dysregulation of inflammatory responses and the production of specific autoantibodies [1].

Several studies have reported elevated levels of brain-reactive autoantibodies in schizophrenic (SZ) patients, including antibodies targeting neurotransmitter receptors [2]. Additionally, investigations have explored the presence of non-organ-specific autoantibodies, such as antinuclear and antiphospholipid antibodies [3], which are often elevated in autoimmune diseases, even when organ-specific autoantibodies are absent.

Antiphospholipid antibodies (aPLs) have garnered significant attention in recent years due to their frequent association with thrombotic events, pregnancy-related complications, and psychiatric symptoms [4]. The aPL antibodies are key markers for antiphospholipid Syndrome (APS), an autoimmune disorder characterized by an increased risk of vascular thrombosis and obstetric complications. APS can be classified as primary when it occurs without an underlying autoimmune disease or secondary when associated with another autoimmune condition, typically systemic lupus erythematosus (SLE). A third subtype, catastrophic APS, is a rare and life-threatening form characterized by the acute onset of thromboses affecting multiple organs [5]. These subtypes vary in clinical severity and management strategies.

The aPLs comprise a heterogeneous group of autoantibodies directed against phospholipids, phospholipid-binding proteins, or their complexes [6], which play a key role in the coagulation process. The aPLs have been shown to interact with cell membranes within specialized microdomains enriched in cholesterol and glycosphingolipids, termed lipid rafts, that are implied in signal transduction pathways. Anti-β2GPI antibodies (anti-β2GPI) react with β2GPI but also annexin A2 and toll-like receptors (TLR2, TLR4) within lipid rafts, triggering proinflammatory and procoagulant signaling pathways in endothelial cells, monocytes, and platelets. These interactions lead to the release of tissue factor (TF) and TNF-α, promoting thrombosis and inflammation, which are central to the pathophysiology of APS [5]. Among the most commonly reported aPLs involved in thrombotic vascular events, particularly in autoimmune disorders, are anticardiolipin antibodies (aCL), which are known to disrupt various cellular and circulatory functions [7].

In schizophrenia, abnormalities in membrane phospholipids have been identified as a key feature [8,9], supporting the hypothesis that schizophrenia may be a metabolic disorder [10,11]. Another theory points to abnormalities in coagulation pathways, particularly reduced tissue plasminogen activator (tPA) activity [12], suggesting a possible pathogenic role for aPLs in the disease. These hypotheses, along with genetic and environmental factors, appear interconnected, contributing to the pathogenesis of this clearly multifactorial disorder.

β2GPI is a plasma glycoprotein involved in coagulation and fibrinolysis [13]. In vitro, β2GPI exhibits several anticoagulant properties [14]. Although its natural function remains unclear, it may contribute to regulating fibrinolysis [15] and platelet activity [16], with modest anticoagulant effects that are amplified when bound to antibodies [17]. β2GPI has also been shown to participate in the phagocytosis of apoptotic neurons and vascular injury in experimental brain stroke [18].

In this study, we report the prevalence of IgG, IgA, and IgM antibodies against both CL and β2GPI in drug-free SZ patients compared to healthy controls, along with the correlation between aPL levels and psychopathology scores.

## 2. Materials and Methods

### 2.1. Subjects

#### 2.1.1. Schizophrenic Patients

Eighty patients who met the DSM-IV diagnostic criteria for schizophrenia [19] were recruited from the Psychiatry Department of Farhat Hached Hospital in Sousse, Tunisia. Each patient underwent a diagnostic assessment through structured clinical interviews using the Structured Clinical Interview for DSM-IV [20]. All patients were in the acute phase of the illness and were either medication naïve (first onset) or had been off medication for at least three months. None of the patients in our cohort had a documented history of thrombosis or autoimmune diseases.

Psychopathological assessments were standardized and conducted by a trained psychiatrist, employing the Brief Psychiatric Rating Scale (BPRS), the Positive and Negative Syndrome Scale (PANSS), the Scale for the Assessment of Positive Symptoms (SAPS), and the Scale for the Assessment of Negative Symptoms (SANS). Additionally, the Clinical Global Impressions (CGI) scale and the Global Assessment of Functioning (GAF) were used to evaluate disease severity and functional status, respectively.

Only patients with a BPRS score ≥ 40 were included in the study. Patients with a history of autoimmune diseases or clinical indications of such disorders were excluded. All participants provided informed consent after receiving a full explanation of the study’s procedures. The study protocol was approved by the Institutional Review Board and the Hospital Ethics Committee.

#### 2.1.2. Healthy Controls

Eighty healthy controls were recruited from consenting blood donors at the Sousse Farhat Hached Hospital. Subjects with a diagnosed autoimmune disease or a history of autoimmune disorders were excluded. All controls were free from any psychotic disorders, which was confirmed by a trained psychiatrist using the Mini International Neuropsychiatric Interview (MINI-Plus). Comprehensive medical histories, physical examinations, and laboratory tests were obtained for both patients and healthy controls.

### 2.2. Antibody Assays

#### 2.2.1. IgG, IgA, and IgM Anti-Cardiolipin Antibodies (aCL)

Serum levels of IgG, IgA, and IgM aCL were measured using a commercial enzyme-linked immunosorbent assay (ELISA) (Orgentec Diagnostika^®^, Mainz, Germany). Results were expressed in arbitrary units, with positivity defined as ≥10 U/mL for IgA and IgG, and ≥7 U/mL for IgM, based on the manufacturer’s instructions.

#### 2.2.2. IgG, IgA, and IgM Anti-beta-2 Glycoprotein-I Antibodies (aβ2GPI)

IgG, IgA, and IgM aβ2GPI levels were determined using a commercial ELISA (Orgentec^®^) with purified human β2-glycoprotein I as the antigen. Results were expressed in arbitrary units, with positivity defined as ≥8 U/mL according to the manufacturer’s guidelines.

### 2.3. Statistical Analysis

The frequencies of aCL and aβ2GPI were compared using Chi-square or Fisher’s exact tests. A *p*-value of less than 0.05 was considered statistically significant. Pearson’s correlation coefficients were calculated to examine the relationships between aCL and aβ2GPI levels and clinical variables. All statistical analyses were performed using SPSS version 11.01.

## 3. Results

### 3.1. Patients

The clinical and demographic characteristics of the patients are summarized in Table 1. Of the 80 patients, 16.2% (13) were medication-naïve, while 83.7% (67) had been medication-free for at least three months. Upon admission, the mean BPRS score was 53.8 ± 5.8 (mean ± SD). Other assessment scores are presented in the same table.

### 3.2. Healthy Controls

Among the 80 healthy controls, 92.5% (74) were female and 7.5% (6) were male. The mean age of the controls was 21.91 ± 5.95 years (range between 17 and 45 years).

### 3.3. Anti-Cardiolipin Antibodies

The frequency of aCL was significantly higher in SZ patients compared to healthy controls (*p* < 0.05) (Table 2), particularly for the aCL-IgM (*p* = 0.002) and aCL-IgA isotypes (*p* = 0.006) (Table 3). Two out of the 17 aCL-positive patients exhibited all three isotypes simultaneously (Table 4), a finding absent among the healthy controls. Additionally, two other patients presented both aCL-IgM and aCL-IgA concurrently (Table 4).

### 3.4. Anti-beta-2 Glycoprotein-I Antibodies

aβ2GPI were significantly elevated in SZ patients compared to healthy controls (27.5%, *p* < 0.001) (Table 2), with eight of the 22 positive patients showing titers higher than 50 U (Table 4). Analysis of individual isotypes revealed that only the aβ2GPI-IgA isotype was significantly more frequent in SZ patients (*p* < 0.001) (Table 3). Two of the 22 aβ2GPI-positive patients exhibited all three isotypes, while three patients had two isotypes.

### 3.5. Anti-Phospholipid Antibodies

**Figure 1:**
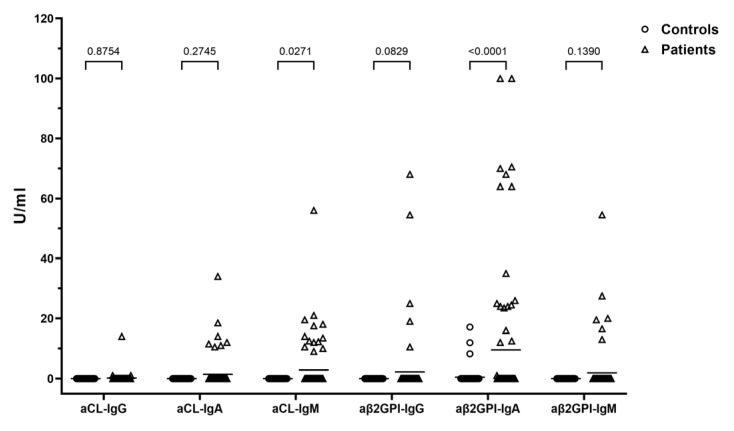
Distribution of aCL (IgG, IgA, and IgM) and aβ2GPI (IgG, IgA, and IgM) in patients and controls.

The frequency of antiphospholipid antibodies (aCL and/or aβ2GPI) was significantly higher in SZ patients (*p* < 0.001) (Table 2). Fourteen SZ patients displayed the simultaneous presence of both aCL and aβ2GPI. Among the 25 patients with aPLs, 18 had the aβ2GPI-IgA isotype (72%), of whom seven exhibited titers exceeding 50 U (Table 4). Additionally, six of the seven aCL-IgA-positive patients had titers above 50 U and were also positive for aβ2GPI-IgA (Table 4). The distribution of aCL (IgG, IgA, and IgM) and aβ2GPI (IgG, IgA, and IgM) in patients and controls is shown in Figure 1.

### 3.6. Correlations Between aCL, aβ2GPI Levels, and Clinical Data

No significant correlations were found between aPL levels and clinical variables such as age, age of onset, clinical form, number of admissions, number of episodes, SAPS, PANSS, SANS, CGI, GAF, or BPRS scores. However, a positive correlation was observed between aCL-IgM levels and disease duration (r = 0.231, n = 80, *p* = 0.039). No significant correlation was found between aCL-IgG or aCL-IgA levels and this parameter.

## 4. Discussion

One of the main findings of our study was the detection of elevated levels of aPLs in chronic SZ patients experiencing an acute exacerbation of illness. These patients were either drug-naïve or had been drug-free for at least three months prior to the study. We found that the prevalence of aPLs (aCL and/or aβ2GPI) was significantly higher in SZ patients compared to healthy controls. Specifically, aCL positivity was observed in 21.25% of patients. This result aligns with previous studies, which reported a higher prevalence of aCL in SZ patients [21,22,23,24,25]. However, contrasting results were obtained by Sirota et al., who found significantly reduced aCL levels in first-episode drug-naïve SZ patients and chronic patients with acute exacerbations after being drug-free for at least two months [26].

Previous research noted increased levels of aCL, particularly of the IgM isotype, in chronic SZ patients undergoing treatment with chlorpromazine, clozapine, or lithium [27,28,29]. More recent studies have suggested that elevated aCL levels are characteristic of both medicated and unmedicated SZ patients, supporting the idea that aCL may not solely be drug-induced [23,24,25]. Our findings, which show significantly elevated aCL-IgM levels in unmedicated SZ patients, corroborate this hypothesis. The pathophysiological significance of elevated aCL-IgM in unmedicated SZ patients remains unclear, particularly given the exclusion of conditions typically associated with raised aCL levels, such as autoimmune diseases, from this study.

Regarding the IgG isotype, previous studies have shown that aCL-IgG is significantly more frequent in SZ patients compared to controls [23,24,25,27,29]. In contrast, our study found no significant difference in aCL-IgG between SZ patients and controls. This discrepancy may be due to several factors, including differences in genetic background, disease heterogeneity, study design, or assay methodology used for aCL detection. Firer et al. suggested that while aCL-IgG reflects a heightened specific immune response in some families with SZ, aCL-IgM is more closely linked to disease development [24]. However, the lack of a statistically significant difference in aCL-IgG between patients and controls in our study suggests that aCL-IgG alone may be insufficient to differentiate between affected and non-affected individuals. A larger-scale study may be necessary to explore the relationship between aCL-IgG positivity and SZ.

Disease heterogeneity might also explain these divergent results. Schizophrenia is increasingly recognized as a spectrum of disorders rather than a single disease entity. Our study excluded patients with clinical or laboratory evidence of conditions known to influence immune function, such as autoimmune diseases, drug-induced lupus, or antiphospholipid syndrome. This criterion was not consistently applied in previous studies. Additionally, variations in laboratory techniques, such as the use of bovine cardiolipin antigens in some studies [23,29], may account for the differences in aCL prevalence. Our study, by contrast, exclusively used human antigens and separately analyzed cardiolipin and β2GPI, with a particular focus on IgA isotypes. Sample size may also be a factor, as our patient cohort was larger than those in some other studies [21,22,23,29].

A key novel finding of our study was the high prevalence of the IgA isotype of aCL in patients with SZ, a result that, to our knowledge, has not been previously reported. Elevated aCL-IgA levels may indicate autoimmune reactivity or contribute directly to the disruption of phospholipid metabolism in SZ. The co-occurrence of aCL-IgM and aCL-IgA observed in our study suggests a potential link between SZ and APS despite the lack of statistically significant aCL-IgG levels. In line with our results, aCL-IgA is generally associated with consensus aPL positivity [30]. Additionally, aCL-IgA has demonstrated lower sensitivity for APS compared to aβ2GPI-IgM or IgA [31], which is why its clinical significance has been questioned. However, isolated positivity for IgA aCL, although rare, has been associated with both venous and arterial thrombosis, highlighting the potential clinical implications of this isotype [32]. Nonetheless, further research into the role of the IgA isotype of aCL antibodies is warranted.

Regarding β2GPI, we found a significantly higher prevalence of aβ2GPI in SZ patients compared to controls, particularly for the IgA isotype (22.5% vs. 1.25%, *p* < 0.001). To our knowledge, this is the first study to separately examine aβ2GPI isotypes in SZ patients. β2GPI, a 50-kDa phospholipid-binding plasma protein, has been implicated in several coagulation pathway functions [14], though its precise role remains unclear. It is well-established in APS, where high titers of aβ2GPI are strongly associated with thrombosis [14,15]. Although none of our patients exhibited clinical features of APS, close follow-up of those with high aβ2GPI titers is recommended.

The high prevalence of aβ2GPI-IgA antibodies in our study is particularly striking, given that IgA antibodies are not yet included in the APS classification criteria. While aCL-IgA and aβ2GPI-IgA were included in the Systemic Lupus International Collaborating Clinics (SLICC) classification criteria in 2012 and the 2019 EULAR/ACR classification criteria [33,34], they remain excluded from the APS classification criteria in the 2023 ACR/EULAR guidelines [35]. Historically, insufficient scientific evidence has contributed to their exclusion from APS criteria [31,32]. However, recent studies have demonstrated that circulating immune complexes of IgA and β2GPI in patients with APS symptoms and isolated aβ2GPI-IgA [36,37]. These circulating immune complexes have been reported to be strongly correlated to thrombotic events, indicating a pathogenic role [37]. Additionally, animal models have provided further evidence of the pathogenicity of aβ2GPI-IgA. Mice inoculated with purified aβ2GPI-IgA, as for IgM and IgG isotypes, developed significantly larger and more persistent thrombi compared to control mice [38].

Our findings, along with emerging research, support the hypothesis that IgA isotypes may hold clinical relevance, particularly in patients with neuropsychiatric symptoms. For instance, aβ2GPI-IgA has been identified as an independent risk factor for ischemic stroke—the most common and severe arterial thrombotic event in APS [39,40]—as well as for acute myocardial infarction and atherosclerotic disease in non-APS populations [32]. These observations raise important questions about the relevance of aβ2GPI-IgA in neuropsychiatric disorders, such as schizophrenia (SZ), and suggest a potential overlap between autoimmune processes and SZ.

Naranjo et al. demonstrated that aβ2GPI-IgA was the most prevalent among both classic and non-criteria aPLs, with 82% of aβ2GPI-IgA-positive patients testing negative for other aPLs [39]. This highlights the diagnostic utility of aβ2GPI-IgA in identifying a subset of patients with seronegative APS [31]. Furthermore, the task force of the 13th International Congress on APS recommended testing for IgA anti-B2GPI in patients meeting APS clinical criteria but lacking other consensus antibodies [32,33,34,35,36,37,38,39,40,41]. Notably, aβ2GPI-IgA has been found to be more common in primary APS and arterial thrombosis, underscoring its potential diagnostic value [31].

Consensus aPLs are aCL-IgG, aCL-IgM, aβ2GPI-IgG, aβ2GPI-IgM, and lupus anticoagulant. We acknowledge the limitation of not performing lupus anticoagulant determination in our study. Many seronegative APS patients are positive for non-criteria aPLs [42]. In addition to aβ2GPI-IgA and aCL-IgA, various autoantibodies have been identified within the non-criteria aPL group, including antibodies against the domain I of β2-GPI (aDI), anti-prothrombin, anti-phosphatidylserine, anti-phosphatidylserine-prothrombin (aPS/PT), annexin V, annexin II, phosphatidylinositol, phosphatidylethanolamine, phosphatidic acid, the vimentin-cardiolipin complex, anti-protein S/protein C, and anti-S100A10 [42,43]. aβ2GPI-IgA, aDI, and aPS/PT have gained significant attention due to their roles in thrombotic events and pregnancy complications [42]. Increasing evidence has been reported on the different antigenic targets of aPLs and their association with thrombosis and pregnancy morbidity, potentially bridging the gap between APS and seronegative APS [42,43].

In conclusion, the elevated levels of aPLs, particularly aCL-IgM and aβ2GPI-IgA, in SZ patients point to a potential autoimmune component in the pathophysiology of SZ. The presence of both aCL and aβ2GPI in some patients, along with the co-existence of multiple isotypes, highlights the need for further investigation to elucidate their role in SZ. Future research should also explore potential correlations between the aPL levels and clinical features of SZ, with larger sample sizes and accounting for genetic factors, disease heterogeneity, and standardized methodologies.

## Figures and Tables

**Table 1:** Demographic and clinical data of schizophrenic patients.

	Schizophrenic Patients (n = 80)
Sex (male/female)	50/30 (62.5%/37.5%)
Age (years)	37.94 ± 1.37 (16–68)
Age of onset (years)	25.03 ± 6.83 (13–44)
Disease duration (years)	12.79 ± 9.47
Family history (yes/no)	42/38 (52.5%/47.5%)
Medication status on admission Medication-naive Medication-free	13 (16.25%) 67 (83.75%)
Subtypes Paranoid Undifferentiated Disorganized	11 (13.75%) 40 (50%) 29 (36.25%)
Psychopathology score ^1^ BPRS at admission PANS at admission SAPS at admission SANS at admission CGI at admission EGF at admission	53.8 ± 5.8 78.4 ± 11.77 37.22 ± 11.69 35.92 ± 12.88 4.72 ± 0.65 37.22 ± 5.35

^1^ The psychopathological scores were based on the Brief Psychiatric Rating Scale (BPRS) and SAPS (Scale for the Assessment of Positive Symptoms), PANS (Positive and Negative Syndrome Scale), SANS (Scale for the Assessment of Negative Symptoms), CGI (Clinical Global Impressions) and EGF (Evaluation Globale du Fonctionnement) scores.

**Table 2:** Frequency of aCL, aβ2GPI, and aPL in schizophrenia patients and healthy controls.

	Schizophrenic Patients (n = 80)	Healthy Controls (n = 80)	*p*
Anti-cardiolipin	21.25% (17/80)	3.75% (3/80)	0.0008
Anti-β2-glycoprotein I	27.5% (22/80)	1.25% (1/80)	<10^−3^
Antiphospholipid	31.25% (25/80)	5% (4/80)	<10^−3^

**Table 3:** Prevalence of different isotypes of aCL and aβ2GPI in SZ patients and healthy controls.

	Schizophrenic Patients (n = 80)	Healthy Controls (n = 80)	*p*
aCL-IgG	3.75% (3/80)	1.25% (1/80)	NS
aCL-IgA	8.75% (7/80)	0%	0.006
aCL-IgM	16.25% (13/80)	2.5% (2/80)	0.002
aβ2GPI-IgG	6.25% (5/80)	0%	NS
aβ2GPI-IgA	22.5% (18/80)	1.25% (1/80)	<10^−3^
aβ2GPI-IgM	7.5% (6/80)	0%	NS

**Table 4:** Titer of aCL and aβ2GPI in SZ patients.

Patient N°	Sex	Anti-Cardiolipin (U/mL)			Anti-β2glycoprotein I (U/mL)		
		IgG	IgA	IgM	IgG	IgA	IgM
1	F	-	-	-	-	16	-
2	M	-	-	12.5	-	-	13
3	F	-	-		-	24	-
4	M	-	12	21	-	68	20
5	F	14	-	-	54.5	24	-
6	M	-	-	9.5	-	23.5	-
7	M	-	11.5	-	-	70	-
8	M	-	-	-	-	12	-
9	M	-	-	10	-	20	-
10	M	-	-	12	-	-	-
11	F	26	34	18	68	>100	16.5
12	F	11	18.5	56	19	>100	54.5
13	M	-	-	9	-	-	-
14	F	-	-	-	-	35	-
15	F	-	-	12.25	-	-	-
16	F	-	-	10.5	-	12.5	-
17	F	-	-	-	-	64	
18	F	-	14	14	10.5	70.5	-
19	F	-		13.5	-	24.5	-
20	M	-	-	-	-	26	-
21	M	-	-	17.5	-		19.5
22	M	-	11	-	-	-	27.5
23	M	-	-	-	25	-	-
24	M	-	-	-	-	25	-
25	M	-	10.5	-	-	64	

## Data Availability

The data presented in this study are available on request from the corresponding author.
